# Identification and analysis of diagnostic senescence-related gene signatures for acute myocardial infarction based on multi-omics data and machine learning

**DOI:** 10.3389/fimmu.2026.1775092

**Published:** 2026-06-05

**Authors:** Hanmo Zhang, Hongyu Huang, Zhuo Jiang, Shuangqi Qian, Xiandu Jin, Zeyan Peng, Fan Huang, Peipei Li, Liping Wei, Zhi Qi, Xin Qi

**Affiliations:** 1 School of Medicine, Nankai University, Tianjin, China; 2 Department of Cardiology, Tianjin Union Medical Center, The First Affiliated Hospital of Nankai University, Tianjin, China; 3 Department of Cardiology, Jilin Provincial Cardiovascular Research Institute, China-Japan Union Hospital of Jilin University, Changchun, China

**Keywords:** acute myocardial infarction, cellular senescence, diagnostic biomarkers, machine learning, multi-omics, neutrophils

## Abstract

**Background:**

Acute myocardial infarction (AMI) incidence increases with population aging, alongside heightened cellular senescence; however, clinically useful senescence-related genes (SRGs) for AMI remain poorly defined. This study aimed to identify AMI-associated SRGs and develop multi-dimensional models for diagnostic evaluation and patient stratification.

**Methods:**

Transcriptomic datasets comprising 106 AMI patients and 76 controls were integrated for differential expression and weighted gene co-expression network analyses. An external independent validation cohort including 37 AMI cases and 15 controls was used for further evaluation. Four machine learning algorithms were applied to identify diagnostic SRGs. The selected genes were validated across bulk RNA-seq, single-cell RNA-seq, proteomic data, and mouse myocardial infarction models. Based on these genes, we constructed three SRG-based models: a diagnostic classifier, a patient stratification system, and a senescence scoring system.

**Results:**

Thirteen AMI-associated SRGs were identified, among which four genes, FOS, SOD2, MXD1, and GRN, showed consistent diagnostic relevance across datasets. The four-gene diagnostic model achieved an AUC of 0.808 and showed favorable clinical net benefit. Patient stratification identified a low-senescence group enriched in anti-inflammatory cells and a high-senescence group characterized by pro-inflammatory neutrophil infiltration. The senescence score showed a moderate positive correlation with neutrophil infiltration.

**Conclusion:**

This study identifies FOS, SOD2, MXD1, and GRN as candidate AMI-associated senescence-related biomarkers and establishes preliminary SRG-based models for AMI diagnosis and stratification. These findings suggest that neutrophil-enriched inflammatory responses are associated with senescence-related transcriptional features in AMI and provide a framework for future studies on senescence-related pathways in personalized AMI management.

## Graphical Abstract

**Figure:**
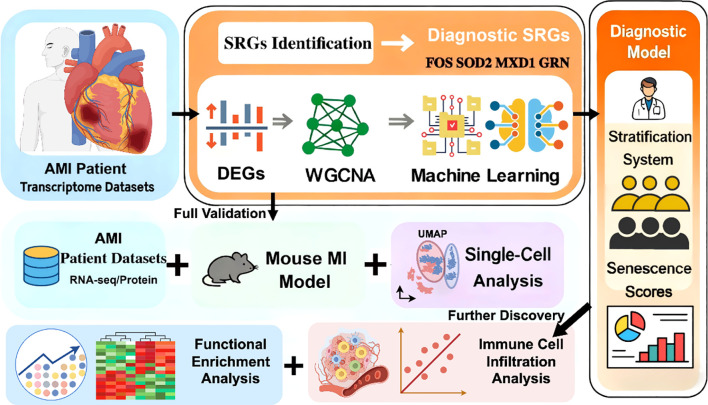

## Introduction

Acute Myocardial Infarction (AMI) is a serious cardiovascular condition with high rates of morbidity, disability, and mortality, significantly impacting global public health, especially with the growing aging population (1, 2). AMI generally results from the sudden blockage of coronary arteries, causing myocardial ischemia and necrosis. Although emergent reperfusion therapies like percutaneous coronary intervention and thrombolytic therapy have advanced considerably, the prognosis for AMI patients remains suboptimal (3). Many patients continue to face risks of complications like heart failure and arrhythmias after the acute phase, highlighting the urgent need to develop novel diagnostic biomarkers for improving clinical outcomes (4).

Cellular senescence, an irreversible state of cell cycle arrest, is linked to numerous age-related diseases (5). Senescent cells accumulate in damaged tissues and secrete proinflammatory factors, resulting in chronic inflammation and impaired tissue function (6). Recent research indicates that various stressors, such as oxidative stress, DNA damage, and inflammation, can induce cellular senescence, which are frequently observed in ischemic myocardium (7). Senescent cells express specific markers and secrete a range of factors (including cytokines, chemokines, and matrix metalloproteinases), which regulate the local tissue microenvironment and influence the behavior of neighboring cells. This process may exacerbate myocardial injury and hinder the repair process.

Recent studies suggest that cellular senescence plays a role in the development and progression of AMI (8). Studies have found that in AMI, oxidative stress and DNA damage in myocardial cells increase significantly, leading to a marked upregulation in the expression of cellular senescence markers (e.g., p16INK4a) (7). These senescent cells accumulate in the damaged myocardium and secrete proinflammatory factors, further intensifying the inflammatory response and tissue damage. Other studies have also indicated that oxidative stress and DNA damage caused by ischemia-reperfusion injury are key factors inducing cellular senescence in AMI (9). Current research mainly describes the phenotype of cellular senescence, with limited systematic studies on the specific role of senescent cells in AMI (10). In particular, the identification and validation of senescence-related genes (SRGs) with diagnostic value in AMI, as well as the clinical application value of these genes in AMI, remain unclear. Importantly, cellular senescence as a biological process should be distinguished from senescence-related gene expression as a transcriptomic readout. Cells expressing senescence-associated genes are not necessarily bona fide senescent cells, and senescence-related transcriptional signatures should therefore be interpreted as biomarker-oriented molecular features associated with senescence-related biology rather than direct proof of senescence status. In this study, we focused on identifying AMI-associated senescence-related transcriptional signatures with potential diagnostic relevance, rather than directly defining senescent cells at the tissue or single-cell level.

This study investigated senescence-related transcriptional features in AMI, identified senescence-related genes, and evaluated their potential diagnostic relevance. In addition, we constructed a novel diagnostic model, a patient stratification system, and a senescence-related scoring system for AMI. We investigated the crucial roles of immune cells in senescence to offer new insights into the immunoregulatory mechanisms of AMI.

## Methods

### Data acquisition and preprocessing

Transcriptome datasets associated with AMI were obtained from the Gene Expression Omnibus database (https://www.ncbi.nlm.nih.gov/geo/), including GSE34198 (49 AMI cases, 48 controls), GSE48060 (31 AMI cases, 21 controls), GSE60993 (26 AMI cases, 7 controls), and GSE29111 (37 AMI cases, 15 controls). Subsequent missing value handling and normalization were performed: in the expression matrix, rows (genes) and columns (samples) with a missing value (NA) proportion exceeding 50% were removed. The impute.knn function from the R package “impute” (11) was employed to fill in missing values using k-nearest neighbors, followed by log2(x + 1) normalization of the data. The datasets GSE34198, GSE48060, and GSE60993 were combined, and batch effects were removed using the removeBatchEffect function from the R package ‘limma’ (version 3.42.2) (12), producing a unified expression profile for the training set.

Protein expression profiles for 48 AMI cases and 50 controls were obtained from the iProx database (https://www.iprox.cn/page/home.html) under accession PXD062794 (13), followed by missing value imputation and normalization.

The senescence gene set (**Supplementary Table 1**, n = 307) used in this study was downloaded from the CellAge database (https://genomics.senescence.info/cells/).

### Differential expression gene analysis

Differential expression gene (DEG) analysis was performed using the “limma” package. Differentially expressed genes were identified using a threshold of |fold change (FC)| ≥ 1.2 and *P* < 0.05. Notably, the selection of |FC| ≥ 1.2 was based on comprehensive consideration of transcriptomic analytical practices, statistical principles, and the biological characteristics of peripheral blood samples in acute myocardial infarction. There is no universal fixed standard for FC threshold selection in transcriptomic studies, which is often determined by research context and objectives. FC reflects the magnitude of expression difference (effect size), while p-value evaluates statistical significance; the combined use of FC and statistical significance helps balance biological effect and statistical reliability. A moderate FC threshold (|FC| ≥ 1.2) facilitates the retention of biologically meaningful genes with moderate expression changes, which is particularly suitable for the “low-amplitude, polygenic synergistic regulation” expression pattern commonly observed in peripheral blood transcriptome studies. To further reduce potential noise and ensure result robustness, the differentially expressed genes were subsequently subjected to multi-step screening including weighted gene co-expression network analysis (WGCNA) and four machine learning algorithms. Volcano plots and expression heatmaps were generated using the R packages “ggplot2” (14) and “heatmap” to visualize the identified differential genes.

### Functional pathway enrichment analysis

Enrichment analyses for Gene Ontology (GO) and Kyoto Encyclopedia of Genes and Genomes (KEGG) pathways were performed using the enrichGO and enrichKEGG functions from the “clusterProfiler” package (15). Gene set sizes were constrained between 5 and 5000. Statistical significance was defined as a p-value less than 0.05 and a false discovery rate (FDR) below 0.1. The results were visualized as bubble plots using the “ggplot2” package.

### Weighted gene co-expression network analysis

The gene co-expression network was constructed, and modules associated with AMI were screened using the R package “WGCNA” (16) was used to construct a gene co-expression network and screen modules associated with AMI. The specific steps were as follows: first, genes with a small median absolute deviation (approximately 50%) were excluded. The pickSoftThreshold function was used to select the soft threshold (β = 16), with the selection criterion of a fitting index > 0.8 (at β = 16, the scale-free topology fit index reached R² = 0.86, and the mean connectivity was 0.11; **Supplementary Figures 1A, B**). The adjacency matrix was derived using the adjacency function and subsequently transformed into a topological overlap matrix (TOM) through the TOMsimilarity function. Gene modules were identified through unsupervised clustering, employing the function ‘hclust’ for hierarchical clustering. The clustering tree branches were segmented into distinct modules using the cutreeDynamic function (**Supplementary Figure 1C**). The mergeCloseModules function was applied to combine modules with highly correlated eigengenes, resulting in four gene modules. To elucidate the biological relevance of the modules, a correlation analysis was conducted between the eigengenes of the four modules and the AMI phenotype, followed by the creation of a module-phenotype association heatmap using the labeledHeatmap function. Gene modules significantly linked to AMI were chosen for further analysis, with module membership and gene significance scores assessed to determine their importance. We selected modules that exhibited significant positive correlations with AMI, as positive associations are more closely relevant to the pathological and diagnostic mechanisms of AMI. Although the correlation coefficients were moderate (r = 0.24 and r = 0.23), both modules showed highly significant statistical significance (*P* < 0.001), supporting their reliability in this peripheral blood transcriptomic study, where sample heterogeneity commonly limits phenotypic correlation coefficients from reaching high levels. Of note, WGCNA was conducted using differentially expressed genes rather than the whole transcriptome. This design reduces noise from low-variation background genes, strengthens disease-relevant signals, and improves the stability of module detection related to AMI.

### Identification of diagnostic SRGs using machine learning

A univariate logistic regression analysis (using *P* < 0.10 as the inclusion criterion) assessed the crude association between each SRG and AMI, with a forest plot illustrating the effect sizes. Four algorithms were independently executed: eXtreme Gradient Boosting (XGBoost) with a learning rate of 0.05 and max depth of 3 using the ‘xgboost’ package (17), Random Forest with 500 trees and 5 variables tried at each split using the ‘randomForest’ package (18), Lasso with 10-fold cross-validation using the ‘glmnet’ package (19), and Gradient Boosting Machine (GBM) with an interaction depth of 3 and shrinkage of 0.05 using the ‘gbm’ package (http://cran.rproject.org/web/packages/gbm/index.html). Variable importance scores were output, and candidate genes were selected according to the “maximum-gap cut-off” strategy based on the ranking of importance scores. The “maximum-gap cut-off” determines the threshold based on the adjacent jumps in the ranked importance scores, with no subjective presets, which can reduce overfitting and retain the most informative variables. Finally, the SRGs screened by each algorithm were intersected to obtain common genes, which were defined as diagnostic SRGs. To evaluate model stability and avoid overfitting, 10 fold cross validation was performed for each machine learning algorithm. The mean AUC, standard deviation (SD), and 95% confidence interval (CI) across all folds were calculated and reported. The mean AUCs of XGBoost, Random Forest, Lasso, and GBM were 0.810 (SD = 0.025), 0.796 (SD = 0.027), 0.775 (SD = 0.030), and 0.803 (SD = 0.028), respectively. All models showed small SD and narrow 95% CI, indicating good generalization ability and low overfitting risk.

### Construction and validation of the diagnostic model

A binary multivariate logistic model was fitted using glm (family = binomial) (20), and the unstandardized coefficient β of each SRG was extracted as the weight. The combined dataset (GSE34198, GSE48060, and GSE60993) was randomly divided into training set and test set according to a ratio of 7:3, and the diagnostic model construction and model validation was used respectively. Linear predictors were calculated, and the rmda:: decision_curve function evaluated the net benefit rate across a threshold range of 1%–99% using these predictors.

A nomogram was constructed using the “rms” package (v6.7.0) (21). In the nomogram, each gene is assigned a score, and the cumulative score of the SRGs predicts an individual’s AMI risk probability. A calibration curve was plotted to verify the consistency between predicted probabilities and observed results.

A risk score was constructed based on the regression coefficients (β_*i*_) of the above model. The risk was calculated using the formula: Risk = Σ β_*i*_ × Expr_*i*_, where Expr_*i*_ denotes the standardized expression level of the *i*-th SRG, and β_*i*_ is the associated regression coefficient. The risk score for each sample was calculated by summing the products of each gene’s expression level and its corresponding regression coefficient. This risk score represents a supervised modeling output derived from cross-sample expression patterns, primarily designed to quantify the discriminative capacity of the model for distinguishing AMI from control samples. As a linear combination of gene expression weighted by regression coefficients, it emphasizes predictive performance at the cohort level.

Decision Curve Analysis (DCA) was used to evaluate the risk score. With “All” (all patients diagnosed with AMI) and “None” (all patients diagnosed as normal) as the baselines, the net benefits of the prediction model under the two threshold probabilities were compared to assess the benefit of the risk score in clinical decision-making. The DCA was performed using the “rmda” package (v1.6) (22), and visualization was conducted in combination with the “ggplot2” package (v3.4.4). In parallel, a logistic regression model was constructed with a binary outcome (1 = AMI, 0 = Control) and the SRGs risk score as the predictor. The results demonstrated a statistical association between the risk score and AMI occurrence, indicating a predictive capability of the model. Importantly, the integration of DCA and logistic regression provides complementary evidence from both statistical and clinical perspectives. While logistic regression quantifies the strength and significance of the association between the risk score and disease occurrence, DCA evaluates the net clinical benefit under practical decision-making scenarios. Together, these analyses comprehensively demonstrate that the SRGs-based model is not only statistically reliable but also clinically valuable, thereby overcoming the limitations of relying on a single analytical approach.

Using the SRG diagnostic model, the ‘pROC’ package was employed to generate the Receiver Operating Characteristic (ROC) curve and compute the Area Under the Curve (AUC) (23), considering AMI as the true positive. For the logistic regression diagnostic model, the input variable for ROC analysis was the calculated risk score derived from the model, combined with the corresponding clinical labels (AMI vs. normal). The AUC was calculated based on the combined dataset (GSE34198, GSE48060, and GSE60993).

To further ensure model robustness and minimize the risk of overfitting and data leakage, a 10-fold cross-validation strategy was implemented within the training set. In this process, the training data were partitioned into ten subsets, where in each iteration, 90% of the data were used as the training fold and the remaining 10% as the validation fold. The model was independently trained within each training fold and evaluated on the corresponding validation fold, and the AUC values were averaged across all folds to estimate the stability and generalizability of the model.

### Animal experiments

Eight-week-old male C57BL/6 mice and 18-month-old male C57BL/6 mice were sourced from Henan Skobes Biotechnology Co., Ltd. All procedures received approval from Nankai University’s Institutional Animal Care and Use Committee (IACUC; approval number 2025-SYDWLL-000757). Following a one-week acclimatization to reduce environmental stress, mice were randomly allocated to experimental groups.

For the 8-week-old cohort, infarct-zone cardiac tissues were collected 24 h after LAD ligation for exploratory RNA-seq analysis.

To further evaluate the age-related relevance of the identified genes, an additional 18-month-old cohort underwent the same MI/sham procedure. In this aged cohort, peripheral blood leukocytes and infarct-zone cardiac tissues were collected 24 h after LAD ligation for qPCR and western blot validation.

The myocardial infarction (MI) model was established by ligating the left anterior descending (LAD) coronary artery, a commonly used technique for inducing MI in mice. The mice were randomly assigned to either the Sham group or the MI group. Both groups were anesthetized using isoflurane and positioned supine. After removing the chest hair, the surgical area was disinfected with 75% ethanol. A left thoracotomy was conducted between the 3rd and 4th intercostal spaces to access the heart. The LAD was ligated using a 6–0 silk suture approximately 2–3 mm below the left atrial appendage. Following ligation, the chest was immediately closed, and any remaining air was removed to avoid pneumothorax. The surgical incision was sutured, and sham-operated mice underwent the same procedure without LAD ligation.

Successful induction of MI was verified 24 hours post-surgery via transthoracic echocardiography using a high-resolution small-animal ultrasound system. Heart samples and plasma were collected and stored in liquid nitrogen until further analysis.

### RNA sequencing and analysis

RNA sequencing (RNA-seq) was performed by Majorbio (Shanghai, China). Poly(A)-enriched mRNA was reverse-transcribed and used to construct strand-specific cDNA libraries, which were sequenced on an Illumina NovaSeq 6000 platform. Clean reads were aligned to the Mus musculus reference genome (GRCm38/mm10, NCBI) using HISAT2. Gene expression levels were quantified as fragments per kilobase of transcript per million mapped reads (FPKM) for downstream visualization and expression comparison.

For differential expression analysis, raw read count data were used as input for DESeq2 (24). Infarct-zone cardiac tissues collected 24 h after LAD ligation from sham mice (n = 3) and MI mice (n = 3) were analyzed. Differentially expressed genes were identified using the criteria of |FC| ≥ 1.5 and *P* < 0.05. Volcano plots were generated using ggplot2 and ggrepel (25).

Given the limited sample size, this RNA-seq analysis was intended primarily as exploratory and supportive *in vivo* evidence for the expression trends of the identified genes.

### Real-time quantitative PCR

Peripheral blood was collected from sham and MI mice anesthetized with isoflurane. After red blood cell lysis using RBC lysis buffer (Solarbio, 4 °C, 15 min), the remaining peripheral blood leukocytes were harvested by centrifugation. Total RNA was extracted using TRIzol (Solarbio), and 1 μg of RNA was reverse-transcribed into cDNA. Gene expression was measured using PerfectStart Green qPCR SuperMix (2×, Transgen). The primer sequences are listed in **Supplementary Table 2**.

### Western blot analysis

For protein analysis, hearts were excised 24 h after LAD ligation, and infarct-zone tissues from the anterior wall were carefully dissected on ice. Tissues were snap-frozen and lysed in RIPA buffer supplemented with Complete™, PhosSTOP™, and 1 mM PMSF. Equal amounts of protein (20 μg) were separated by 10% SDS-PAGE, transferred onto a 0.22 μm PVDF membrane, and blocked with 5% skim milk in TBST. Membranes were incubated with primary antibodies overnight at 4 °C, followed by HRP-conjugated secondary antibodies (1:5,000) for 1 h at room temperature. Signals were detected using ECL (Thermo Fisher) on a Tanon 5200 system. Band intensities were quantified using NIH ImageJ and normalized to GAPDH. A total of six independent biological samples per group (Sham n = 6, MI n = 6) were included for western blot validation. The antibodies used are listed in **Supplementary Table 3**.

### Immune cell infiltration analysis

The ImmuCellAI algorithm (26) (https://guolab.wchscu.cn/ImmuCellAI/) was employed to analyze expression profiles and quantify immune cell infiltration abundance.

### Consensus clustering analysis

Consensus clustering analysis was performed using ConsensusClusterPlus (27). Agglomerative pam clustering utilized the 1-Spearman correlation coefficient as the distance metric, conducting 10 repeated samplings on 80% of the samples. The optimal number of clusters was determined based on empirical cumulative distribution function (CDF) plots. Based on the expression profiles of senescence-related genes (SRGs), AMI patients were stratified into distinct molecular subtypes.

Subsequent analyses were conducted at the whole-transcriptome level. Immune infiltration was estimated using the ImmuCellAI algorithm based on predefined immune cell marker genes, and functional enrichment analyses were performed using genome-wide differentially expressed genes between clusters. This analytical framework enables the characterization of subtype-specific biological features beyond the initial clustering features.

### Construction of AMI senescence-related scores

AMI senescence-related scores (ASS) were constructed based on the pre-defined diagnostic SRGs. The gsva() function from the R package “GSVA” (method = “ssgsea”) (28) was used to quantifies the coordinated activation based on the relative expression ranks within each sample. The obtained scores were normalized by z-score to facilitate inter-sample comparison and visualization.

For the ASS-based senescence scoring model, the input consisted of the ASS calculated via ssGSEA and the corresponding clinical labels (AMI vs. normal). The AUC analysis was performed on the combined dataset (GSE34198, GSE48060, and GSE60993). Additionally, a 10-fold cross-validation was performed to evaluate the stability and generalizability of the AUC model. In this process, the training data were partitioned into ten subsets, with 90% of the data used as the training fold and the remaining 10% as the validation fold in each iteration. The model was independently trained within each training fold and evaluated on the corresponding validation fold, with the AUC values averaged across all folds to assess the robustness and generalizability of the model.

ASS represents an unsupervised pathway-level metric that reflects the intrinsic activity of cellular senescence programs, independent of phenotype labels, thereby providing a mechanistically interpretable measure of senescence status.

### Acquisition and analysis of single-cell RNA-seq data

The single-cell transcriptome dataset of AMI (GSE163129, n=5, cell=40,314) was used. Referring to the analysis workflow from the original study (https://github.com/Junglab-CMC/Macrophage-heterogeneity-after-MI), the Seurat tool (29) was employed to process, cluster, and annotate cells, resulting in their classification into nine primary cell types. UMAP was employed to reduce the dimensionality of single-cell gene expression data, enabling visualization of cell distribution and ASS scores in two dimensions.

The single-cell ASS was calculated using the gsva() function from the GSVA R package (version 1.34.0) with parameters: method = “ssgsea”, kcdf = “Gaussian”. This algorithm treats each individual cell as an independent sample and quantifies the enrichment level of the four-gene diagnostic signature via the running-sum algorithm, which integrates gene rank order and coordinated expression patterns. The obtained single-cell ASS values were normalized by z-score to facilitate inter-cell comparison and visualization.

Dot plots were used to show the expression differences of diagnostic SRGs (GRN, MXD1, FOS, SOD2) and ASS scores among different celltypes. The dot size indicates the percentage of cells that express the gene, while the color shows the gene’s average expression level.

### Statistical analysis

Statistical analyses were conducted using R software (v4.2.2). Based on the distribution characteristics of RNA-seq data and the prevalent analytical paradigms in the field of bioinformatics, we employed the Wilcoxon test for the analysis of different groups, followed by multiple testing correction. Spearman’s correlation test was used to calculate the correlation between different variables. Odds ratio (OR) and their 95% confidence intervals were reported. A p-value below 0.05 was deemed statistically significant. *P*-values were categorized as: not significant (ns), *P < 0.05, **P < 0.01, ***P < 0.001, and ****P < 0.0001.

## Results

### Identification of senescence-related genes in AMI

**Figure 1:**
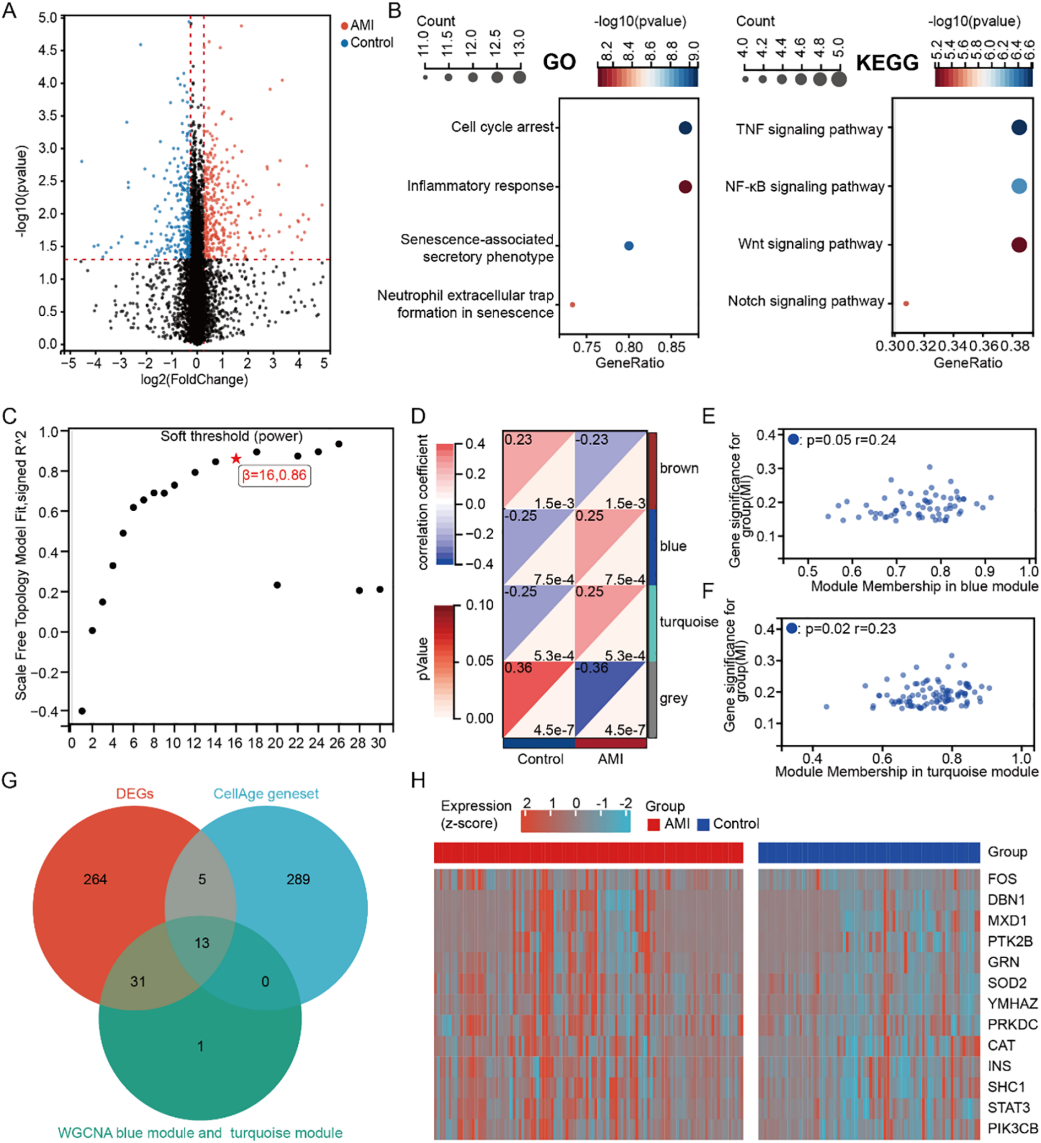
Identification of senescence-related genes in AMI. **(A)** Volcano plot of differentially expressed genes (DEGs). **(B)** Bubble plot of pathway enrichment for DEGs, showing GO and KEGG terms. Rows represent gene ratios and columns represent enriched pathways. **(C)** Soft-threshold screening for WGCNA. Left y-axis: scale-free topology fit R²; right y-axis: mean connectivity. β = 16 was chosen when R² > 0.8 and the curve reached a plateau. **(D)** Module–trait correlation heatmap. Rows: four color modules identified by WGCNA; columns: AMI vs. Control clinical trait. Numbers in cells are Spearman r values; color intensity reflects correlation strength. **(E, F)** Scatterplots of module membership vs. gene significance for **(E)** the blue module and **(F)** the turquoise module. **(G)** SchematicVenn diagram of triple overlap among DEGs, blue + turquoise module genes from WGCNA, and senescence genes from the CellAge database, yielding 13 AMI-associated senescence-related genes (SRGs). **(H)** Heatmap of SRGs expression. Z-score normalized expression of the 13 genes in AMI versus Control groups.

After integrating human AMI transcriptome cohorts (GSE34198, GSE48060, GSE60993) and correcting for batch effects, a total of 313 differential genes were identified by comparing AMI and normal samples (|FC| ≥ 1.2, *P* < 0.05; **Figure 1A**). GO and KEGG analyses indicated that the differentially expressed genes (DEGs) were predominantly associated with pathways related to senescence and inflammation, such as cell cycle arrest, inflammatory response, and TNF signaling pathway. This suggests a link between senescence, inflammation, and the development of AMI (**Figure 1B**). Gene co-expression network analysis was subsequently conducted using the DEGs. The network achieved a scale-free topology with a soft threshold power β of 16, indicated by R² exceeding 0.8 (**Figure 1C**), and 4 stable modules were identified (**Figure 1D**).The blue and teal modules exhibited the strongest correlation with AMI status, with correlation coefficients of 0.24 and 0.23, respectively (**Figures 1E, F**).By intersecting the DEGs, the hub genes of the two modules, and the senescence gene set from the CellAge database, 13 high-confidence senescence-related differential genes (SRGs) were identified (**Figure 1G**; **Supplementary Table 4**), and a heatmap showed the expression of these SRGs (**Figure 1H**).

### Identification of diagnostic SRGs for AMI based on multiple machine learning methods

**Figure 2:**
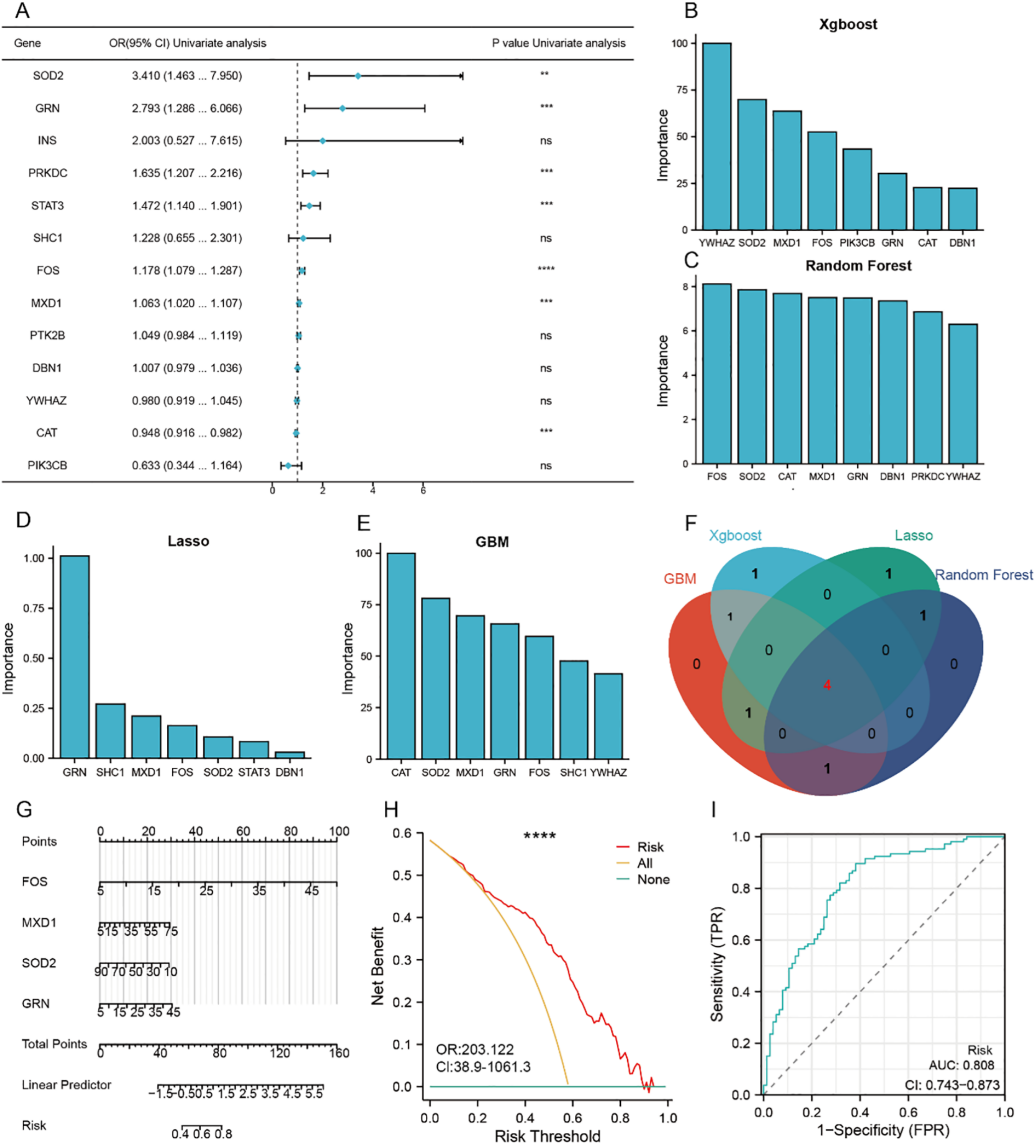
Identification of diagnostic SRGs for AMI based on multiple machine learning methods. **(A)** Forest plot of univariable logistic regression (Wald test) showing the strength of association between each SRG and outcome. **(B–E)** Bar plots of variable importance ranks generated by XGBoost **(B)**, Random Forest **(C)**, Lasso **(D)** and GBM **(E)**. **(F)** Venn diagram identifying the diagnostic SRGs (FOS, SOD2, MXD1, GRN) common to all four algorithms. **(G)** Nomograms constructed from 4 SRGs are used to show the contribution of genes in the model. **(H)** DCA depicting the net clinical benefit of the 4-SRGs-based model across risk thresholds from 1% to 99%, where the P-value, OR, and CI are derived from the logistic regression model constructed with the binary outcome (1=AMI, 0=Control). **(I)** ROC curves of 4-SRGs-based risk score in the combined dataset (GSE34198, GSE48060, and GSE60993), illustrating the discriminative performance of the model for distinguishing AMI from control samples. *P*-values were categorized as: not significant (ns), **P < 0.01, ***P < 0.001, and ****P < 0.0001.

To evaluate the discriminative ability of individual SRGs for AMI, we first conducted univariate logistic regression and plotted a forest plot to visualize the effect sizes. The results showed that higher log-normalized SOD2 expression was significantly associated with increased odds of AMI (OR = 3.410), indicating a positive association between SOD2 expression and the AMI phenotype (**Figure 2A**). To further screen SRGs with universal roles, four algorithms—XGBoost, Random Forest, Lasso, and GBM—were used to identify feature genes with potential diagnostic value for AMI (**Figures 2B–E**). Among these, FOS, SOD2, MXD1, and GRN were the intersecting genes of the four feature gene sets (**Figure 2F**) and were designated as diagnostic SRGs. We developed a diagnostic model using a multivariate logistic approach to assess the clinical significance of SRGs, determining each gene’s weight and calculating the AMI risk score for each sample. A nomogram visually represented the significance of each gene within the AMI diagnostic model (**Figure 2G**). Based on the above integrative analysis, four feature genes (FOS, SOD2, MXD1, and GRN) were ultimately identified, and a diagnostic model was constructed based on this four-gene signature. To validate the performance of the diagnostic model, we conducted a decision curve analysis (DCA). It was found that within a wide range of diagnostic risk score thresholds, the risk score curve was significantly higher than the “All” (all patients diagnosed as AMI) and “None” (all patients diagnosed as normal) curves, indicating that the model had obvious clinical net benefits and showed acceptable discriminatory ability for AMI, with high practical value in clinical diagnostic decision-making (**Figure 2H**). Furthermore, the model’s ROC curve demonstrated an AUC of 0.808 (**Figure 2I**), and these results suggest that the four-gene model has acceptable discriminatory performance and potential utility as a composite diagnostic tool for AMI. We also performed independent validation of the diagnostic model using the external independent dataset GSE29111. The results yielded an AUC value of 0.749 and CI of 0.611-0.886 in the external validation cohort, demonstrating favorable diagnostic performance (**Supplementary Figure 2**).

### Multi-dataset and experimental validation of diagnostic SRGs for AMI

**Figure 3:**
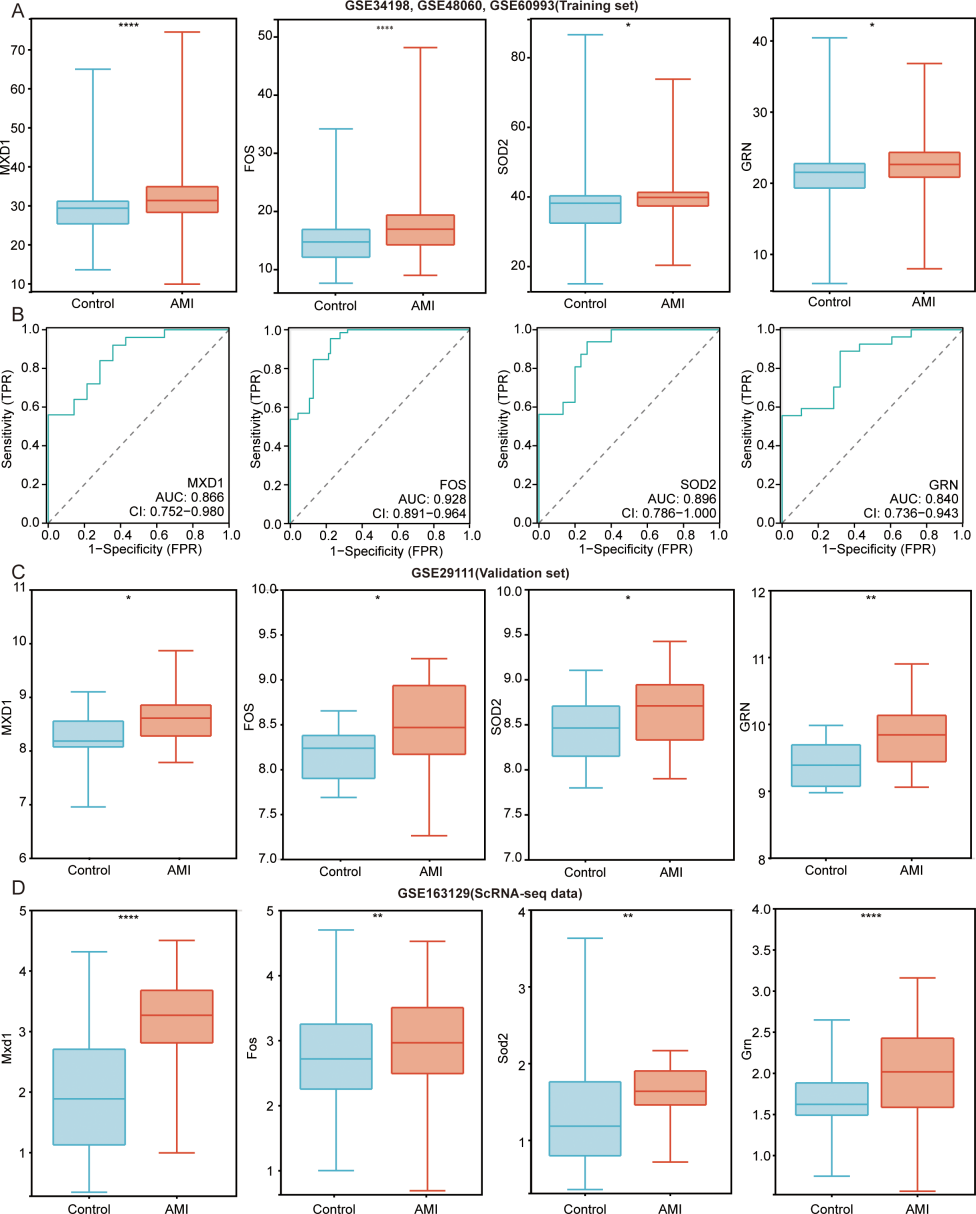
Validation of expression and diagnostic characteristics of 4 diagnostic SRGs in bulk and single-cell RNA-seq data. **(A)** Boxplots of diagnostic SRGs expression levels in AMI versus control groups (Wilcoxon test). **(B)** ROC curves of diagnostic SRGs illustrating their diagnostic efficacy, with data derived from GSE34198, GSE48060, and GSE60993 (Training Set). **(C)** Boxplots of diagnostic SRGs expression levels in the validation cohort GSE29111. **(D)** Boxplots of diagnostic SRGs expression levels in the single-cell validation dataset GSE163129. *P*-values were categorized as: *P < 0.05, **P < 0.01, ****P < 0.0001.

To further validate the diagnostic role of the diagnostic SRGs in AMI, we first confirmed that these 4 genes were consistently highly expressed in the AMI group in the training dataset (**Figure 3A**). We then used GSE29111 as a validation set and found that these genes were also highly expressed in the AMI group (**Figure 3C**). The ROC curves showed that all 4 genes exhibited good diagnostic performance and could effectively distinguish the AMI group from the control group (**Figure 3B**). Analysis of the AMI single-cell dataset GSE163129 revealed significantly elevated expression levels of these four genes in the AMI group (**Figure 3D**). These findings suggest that the identified SRGs may serve as candidate AMI-associated biomarkers with potential diagnostic relevance across multiple datasets.

**Figure 4:**
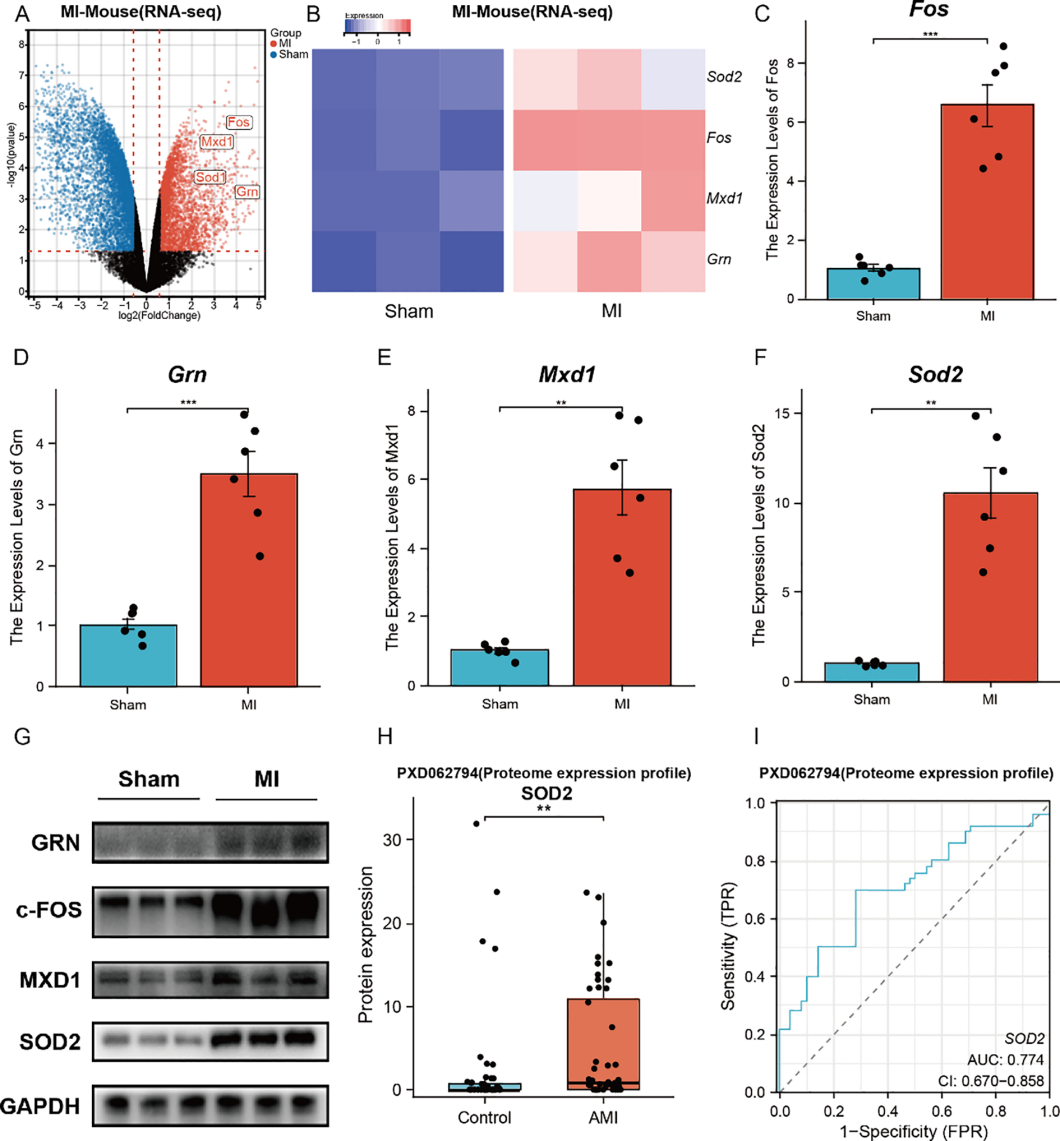
Validation of RNA and protein levels of 4 diagnostic SRGs in AMI. **(A)** Volcano plot of differential expression analysis in mouse cardiac RNA-seq, showing DEGs between the MI and sham groups. RNA-seq was performed using infarct-zone cardiac tissues collected 24 h after LAD ligation (Sham n = 3, MI n = 3). **(B)** Heatmap of commonly differentially expressed SRGs between sham and MI groups. **(C–F)** Relative mRNA expression levels of *Mxd1*, *Fos*, *Sod2*, and *Grn* in peripheral blood leukocytes from 18-month-old sham and MI mice, as determined by qPCR. (n = 6 per group, mean ± SD; Wilcoxon rank-sum test). **(G)** Representative western blots of GRN, c-FOS, MXD1, and SOD2 in infarct-zone cardiac tissues from 18-month-old sham and MI mice. **(H)** Comparison of SOD2 protein abundance between healthy controls and AMI patients. **(I)** ROC curve of SOD2 for discriminating AMI from controls using proteomic data from PXD062794. *P*-values were categorized as: not significant (ns), **P < 0.01, ***P < 0.001.

To further validate the role of the four diagnostic SRGs in AMI, we first performed RNA-seq using infarct-zone cardiac tissues collected 24 h after LAD ligation in 8-week-old sham and MI mice. Differential expression analysis showed that *Fos, Mxd1, Sod2*, and *Grn* were upregulated in the MI group (**Figure 4A**). The heatmap further showed that these four SRGs could distinguish MI samples from sham controls (**Figure 4B**). Given the limited sample size of this young mouse RNA-seq cohort (Sham n = 3, MI n = 3), these results should be interpreted as exploratory and supportive transcriptomic evidence from a standard acute MI model.

To further address the age-related relevance of these findings, we additionally performed validation experiments in 18-month-old mice with MI and age-matched sham controls. qPCR analysis of peripheral blood leukocytes showed that *Mxd1, Fos, Sod2*, and *Grn* were significantly elevated in the aged MI group (**Figures 4C–F**). At the protein level, western blot analysis of infarct-zone cardiac tissues, together with densitometric quantification, confirmed the upregulation of GRN, c-FOS, MXD1, and SOD2 in aged MI mice (**Figure 4G**; **Supplementary Figures 3A–D**). These results provide additional support for the age-related relevance of the identified genes in an *in vivo* MI setting.

Furthermore, analysis of the human peripheral blood proteomic dataset (PXD062794) showed that SOD2 protein abundance was significantly increased in AMI patients (**Figure 4H**), and the ROC curve yielded an AUC of 0.774, supporting its potential diagnostic relevance (**Figure 4I**). However, among the four diagnostic SRGs, only SOD2 had available and analyzable protein expression data in the public plasma proteomic dataset PXD062794. Future studies based on larger-scale or self-established proteomic datasets are needed to verify the protein expression levels of the other three diagnostic SRGs in acute myocardial infarction. In addition, the qPCR and western blot results obtained from 8-week-old mice were retained as supplementary data (**Supplementary Figures 3E–I**), providing supportive evidence that the identified genes also showed concordant dysregulation in a standard acute injury model.

### Immune cell infiltration analysis based on the SRG-based diagnostic model

We utilized the ImmuCellAI algorithm to analyze the relationship between immune cell infiltration and risk scores derived from the SRG-based diagnostic model. Our findings indicated a higher prevalence of anti-inflammatory cells, like iTreg cells, in the low-risk group, while pro-inflammatory cells, including Th17 cells, macrophages, and neutrophils, were notably more abundant in the high-risk group. Neutrophil infiltration was significantly greater in the high-risk group than in the low-risk group (**Supplementary Figure 4A**). Analysis revealed a moderate positive correlation between the risk score and neutrophil infiltration (Spearman R = 0.456, *P* < 0.001), indicating significant recruitment and activation of neutrophils in high-risk patients. The risk score showed a positive correlation with macrophages, Tfh cells, and Th17 cells, while exhibiting a negative correlation with CD8^+^ T cells, exhausted T cells, iTreg cells, Th1 cells, Th2 cells, and NKT cells (**Supplementary Figures 4B–K**, all *P* < 0.001). Given the known involvement of neutrophils in acute inflammation and tissue injury (30), these findings suggest that higher risk scores are associated with increased neutrophil infiltration and a more pro-inflammatory immune microenvironment in AMI. This further validated the effectiveness of the SRG-based diagnostic model in characterizing differences in the immune microenvironment.

### Construction of patient classification system based on SRGs

**Figure 5:**
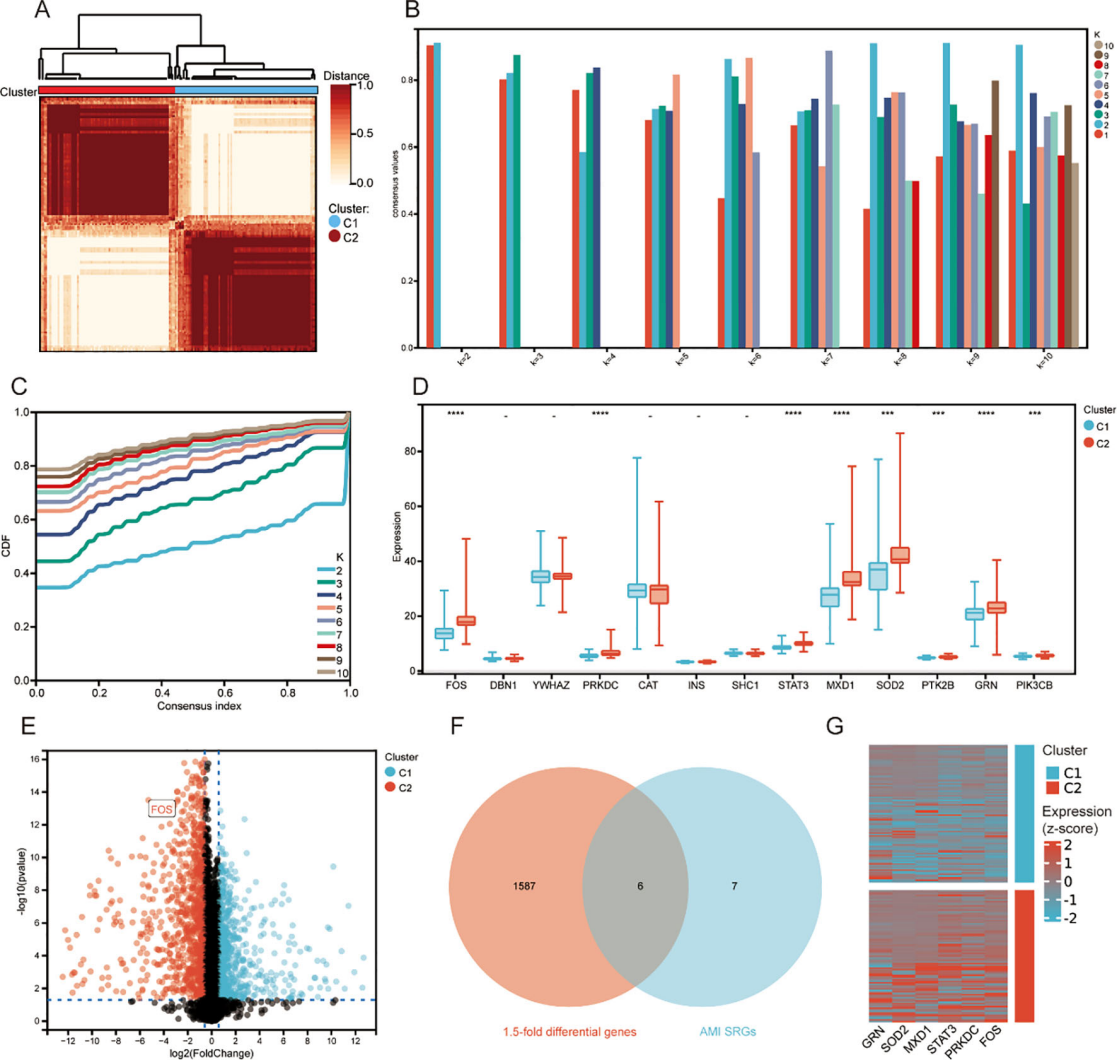
Construction of patient classification system based on SRGs. **(A)** Consensus clustering heatmap showing the identified molecular clusters. **(B)** Consensus clustering bar plot displaying consensus scores for each cluster number. **(C)** Cumulative distribution function (CDF) curve illustrating the stability and consistency of clustering results across different cluster numbers; y-axis: consensus score, x-axis: CDF value. **(D)** Boxplots of SRG expression levels across clusters, revealing expression differences among clusters. **(E)** Volcano plot of DEGs between C1 and C2. **(F)** Schematic Venn diagram depicting the intersection between 1.5-fold DEGs and SRGs, yielding key genes. **(G)** Heatmap of the six key genes showing expression differences between C1 and C2 (z-score). P-values were categorized as: ***P < 0.001, and ****P < 0.0001.

To investigate the role of SRGs in stratifying AMI patients for personalized MI treatment, we employed consensus clustering (**Figure 5A**). When the number of clusters k = 2 (**Figure 5B**), additional clusters yielded only marginal gains (**Figure 5C**). Together, these results strongly support k=2 as the statistically optimal number of clusters. The clustering results showed relatively high consistency and stability. Furthermore, since we found that diagnostic SRGs (FOS, MXD1, GRN and SOD2) were significantly highly expressed in the C2, we initially determined that the C2 was defined as a cluster with high senescence-related signature (**Figure 5D**). Subsequently, through DEGs analysis between C1 and C2, we obtained DEGs (|FC| > 1.5, *P* < 0.05) (**Figure 5E**), and intersected these genes with the SRGs to obtain six key genes (**Figure 5F**). All six key genes exhibited high expression levels within the C2 (**Figure 5G**), we concluded that the C2 exhibited high cellular senescence-related characteristics in AMI patients. We also found that the diagnostic SRGs (FOS, MXD1, GRN and SOD2) were also among the cluster-specific DEGs, which further illustrated the universality of these 4 genes. These results all supported the effectiveness of the diagnostic model based on SRGs, especially diagnostic SRGs, in identifying AMI clusters.

### Immune cell infiltration and functional enrichment analysis based on SRG-based AMI clusters

**Figure 6:**
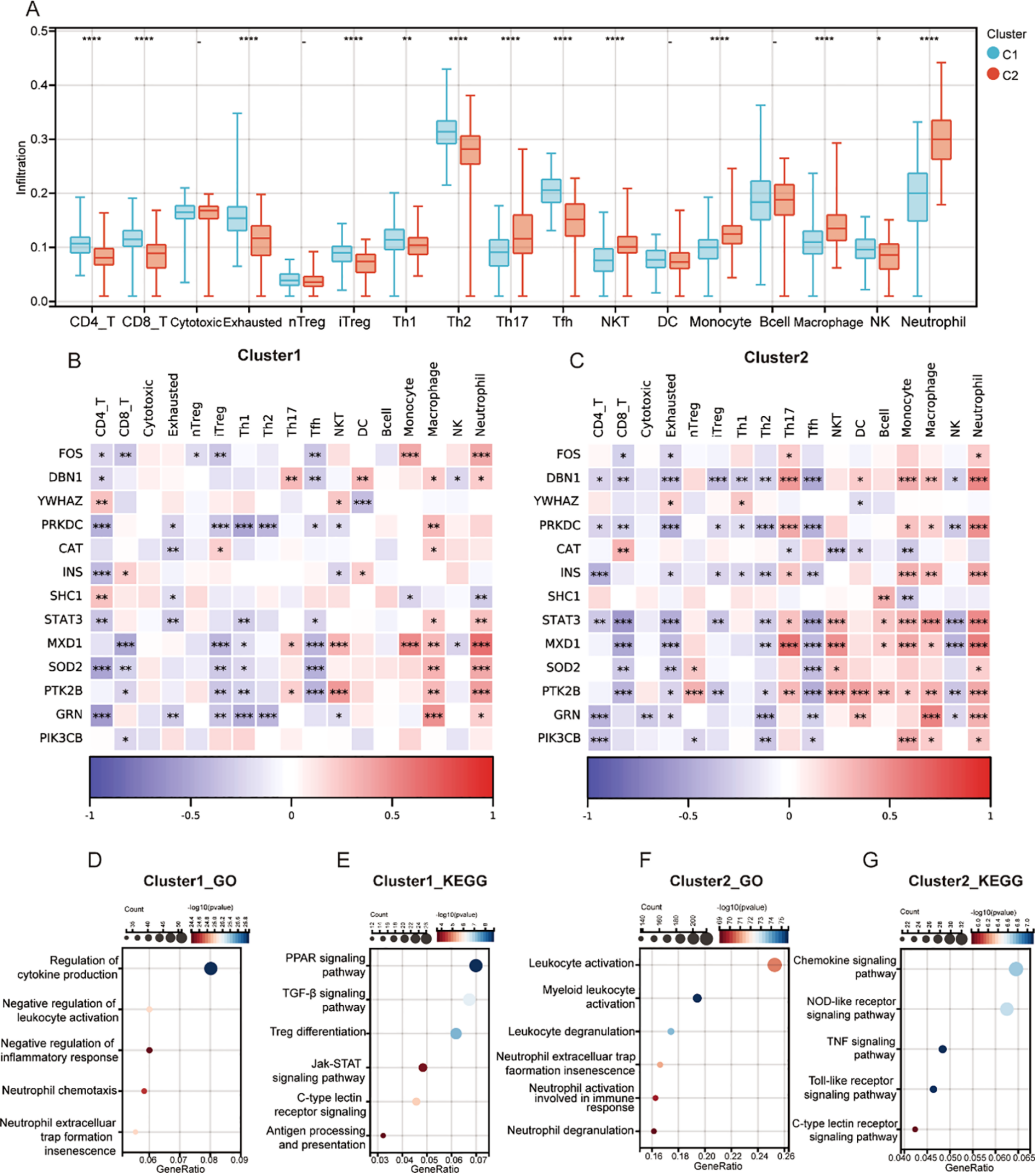
Immune cell infiltration and functional analysis based on SRG-based AMI clusters. **(A)** Boxplots of immune-cell infiltration levels showing differences across AMI clusters. Significance p-values adjusted for multiple comparisons. **(B, C)** Expression-correlation heatmaps between SRGs and individual immune-cell types in C1 **(B)** and C2 **(C–G)** Bubble plots of pathway-enrichment results for each cluster; rows represent gene ratios and columns indicate enriched pathways: GO analysis for C1 **(D)**, KEGG analysis for C1 **(E)**, GO analysis for C2 **(F)**, and KEGG analysis for C2 **(G)**. *P*-values were categorized as: not significant (ns), *P < 0.05, **P < 0.01, ***P < 0.001, and ****P < 0.0001. “cytotoxic” denotes cytotoxic CD8+ T cells, and “exhausted” denotes exhausted CD8+ T cells.

To deeply explore the immune cell infiltration and functional enrichment of AMI clusters, we performed immune infiltration analysis using ImmuCellAI algorithm. The C1 exhibited a high infiltration of anti-inflammatory cells, such as nTreg cells and iTreg cells, whereas the C2 showed a significant increase in proinflammatory cells, including Th17 cells, macrophages, and neutrophils. Neutrophil infiltration was notably higher in the C2 compared to the C1 (**Figure 6A**). The correlation heatmap showed that the correlation between SRGs and various immune cells was generally consistent in both C1 and C2. However, in the C2, the positive correlation between SRGs and some proinflammatory immune cells (e.g., neutrophils) was more significant (**Figures 6B, C**), indicating the immune response is crucial in AMI pathogenesis.

Functional enrichment analyses using GO and KEGG revealed that DEGs in the C1 are predominantly associated with anti-inflammatory pathways, including negative regulation of leukocyte activation, inflammatory response, TGF-β signaling, and Treg cell differentiation (**Figures 6D, E**), whereas the C2 DEGs were associated with proinflammatory pathways, including myeloid leukocyte activation, neutrophil activation, TNF signaling, and Toll-like receptor signaling pathways (**Figures 6F, G**). This suggests that the C2 shows increased inflammation and immune response traits in AMI patients, indicating that targeting cellular senescence could offer novel strategies for AMI prevention and treatment.

### Construction of senescence-related scores and immune cell infiltration analysis in AMI

**Figure 7:**
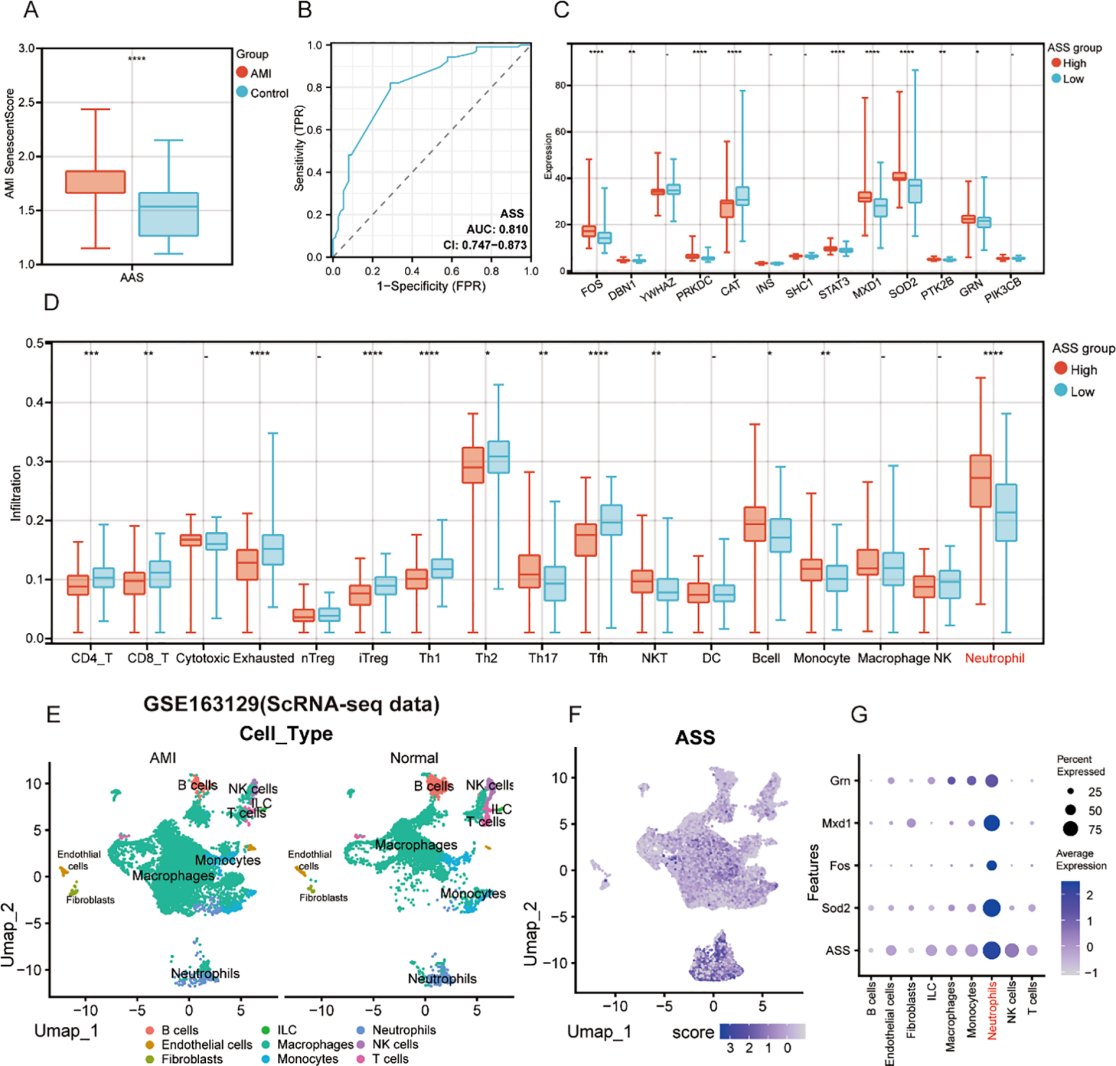
Construction of senescence-related scores and immune cell infiltration analysis in AMI. **(A)** Boxplot of ASS distribution in AMI vs. control samples. **(B)** ROC curve evaluating the diagnostic performance of ASS for AMI. **(C)** Boxplots of SRGs expression levels between high- and low-ASS groups. **(D)** Boxplots of immune-cell infiltration levels comparing high- and low-ASS groups. Significance p-values adjusted for multiple comparisons. **(E)** UMAP projection of major immune-cell types in the GSE163129 scRNA-seq dataset. **(F)** UMAP visualization of ssGSEA-derived ASS scores at single-cell resolution, illustrating score distribution across celltypes. **(G)** Dot plot displaying the expression distribution of GRN, MXD1, FOS, SOD2 and ssGSEA-derived ASS score across immune cells. Dot size indicates the fraction of cells expressing the gene within each celltype; color represents the average expression level. *P*-values were categorized as: not significant (ns), *P < 0.05, **P < 0.01, ***P < 0.001, and ****P < 0.0001. “cytotoxic” denotes cytotoxic CD8+ T cells, and “exhausted” denotes exhausted CD8+ T cells.

We first constructed the ASS based on the diagnostic SRGs and evaluated its diagnostic value in AMI. The ASS represents an alternative manifestation of the same gene set features as the previously constructed diagnostic model, and therefore exhibits a certain degree of similarity in the results. The ASS was significantly higher in AMI samples than in control samples (**Figure 7A**), and the ROC curve showed that it had high discriminative ability for AMI (**Figure 7B**). A comparative analysis of senescence-related gene expression between high and low ASS groups showed a significant upregulation of diagnostic SRGs (FOS, MXD1, GRN, and SOD2) in the high-score group (**Figure 7C**), suggesting that the ASS can effectively characterize the gene features related to senescence. Immune infiltration analysis showed that neutrophils, macrophages, and some T cell subsets were significantly enriched in samples with high ASS, while dendritic cells and B cells showed a decreasing trend (**Figure 7D**), suggesting a strong link between ASS and immune microenvironment activation. The correlation heatmap showed that the correlation between SRGs and various immune cells was generally consistent in both high and low ASS group (**Supplementary Figures 5A, B**). These results indicated that in high-score samples, inflammation-related signals were associated with stronger senescence-related transcriptional features, suggesting a close link between inflammatory activation and senescence-associated signatures in this group.

Single-cell transcriptome data of AMI was used and annotated to further analyze senescence in immune cells (**Figure 7E**), and the ASS scores were significantly higher in proinflammatory cell populations such as neutrophils and macrophages (**Figure 7F**). To corroborate these findings, we analyzed the distribution of ASS signature genes within the single-cell data. The findings indicated that the four core genes were predominantly located in neutrophils, aligning with the immune infiltration analysis trend (**Figure 7G**). These results suggested that, in AMI, senescence-related transcriptional signals are associated with neutrophil activation and pro-inflammatory responses, and that the ASS may reflect an immune microenvironment linked to both inflammatory activation and senescence-related features.

## Discussion

With the aging of the population, the increasing incidence of AMI has attracted growing attention (2). Cellular senescence is a permanent halt in the cell cycle that can lead to persistent inflammation and tissue dysfunction (31). These pathological changes worsen myocardial injury and impede repair, potentially contributing significantly to the development and progression of AMI (32). However, current research on the systematic identification of senescence-related genes in AMI and their clinical translational application remains limited.

This study utilized multi-omics data, including bulk RNA-seq, scRNA-seq, and proteomics, to screen and validate 13 AMI-related SRGs, identifying four core genes (FOS, SOD2, MXD1, GRN) with universal diagnostic significance. These SRGs showed stable consistency across different datasets and experimental models. Although the final number of SRGs obtained was relatively small, this was not due to insufficient statistical power, but rather to the stringent data filtering strategy employed. The analytical framework of this study prioritizes specificity and biological authenticity over extensive gene coverage, which helps reduce false-positive results and ensures the robustness of the identified biomarkers. On this basis, the study constructed three novel models based on SRGs—a diagnostic model, a patient stratification system, and a senescence-related score system—all of which exhibited excellent predictive performance and stratification potential in AMI.

Despite the rapid development of machine learning and multi-omics technologies, which has promoted in-depth identification of diagnostic biomarkers (33) and characteristics of patients with different clusters, this study still has the following innovations:

First, this study integrates differential analysis with WGCNA and employs several machine learning methods to identify diagnostic SRGs for AMI, unlike traditional methods that solely depend on differential expression analysis (34). By intersecting the screening results of different methods, the bias that may be caused by a single algorithm was effectively avoided, and the stability of the diagnostic biomarkers were improved. Subsequently, the study verified the selected biomarkers in bulk RNA-seq, scRNA-seq, proteomics, and mouse models, confirming their high reliability. Compared with a single technical approach, the strategy of this study better ensures the diagnostic reliability of the identified SRGs.

Second, compared with other researches on clinical decision models for AMI (35), our study evaluates patients’ AMI status from multiple aspects of diagnosis, classification, and scoring, which may help improve the reliability of risk stratification. Based on AMI SRGs, the study constructed novel multi-dimensional clinical decision models, whose performance was systematically evaluated using methods such as ROC curves and DCA. To enhance the applicability of the four-gene signature derived from the diagnostic model, we further developed an ASS using the same gene set. The ASS also demonstrated robust diagnostic performance and showed a high degree of concordance with the diagnostic model, suggesting that the coordinated expression pattern of these genes can be effectively captured through alternative scoring strategies. The results showed that the models based on SRGs had favorable diagnostic performance and potential clinical net benefit, suggesting that SRGs have promising reference value in AMI molecular diagnosis and individualized risk assessment, and warrant further investigation for application in the early diagnosis, risk evaluation, and individualized stratified treatment of AMI. Notably, although these analyses were derived from a shared set of SRGs, the risk model, ASS, and molecular subtypes serve distinct purposes, representing pathway activity characterization, diagnostic prediction, and patient stratification, respectively.

Third, the four diagnostic genes identified in this study provide new insights into our understanding of the senescent phenotype in AMI. Specifically, to provide biological insight into our findings, we next explore the potential mechanisms through which FOS, SOD2, MXD1, and GRN contribute to cellular senescence in AMI, emphasizing the sequential process of “stress activation—molecular action—senescent phenotype”. FOS, as a key transcriptional regulator driving the formation of the senescence-associated secretory phenotype (SASP), is rapidly activated as an early response gene under AMI-induced ischemia-hypoxia and inflammatory stimulation, forms an AP-1 transcription complex with JUN, and directly binds to the promoter regions of multiple SASP-related genes (such as IL6 and IL1B), significantly enhancing the transcription and secretion of pro-inflammatory factors under the synergistic effect of DNA damage response (DDR) and NF-κB signaling, thereby promoting the establishment and maintenance of the inflammation-related senescent phenotype (36). SOD2, a key node transitioning from antioxidant defense to a marker of mitochondrial senescence, is upregulated in the early stage of AMI as part of the mitochondrial antioxidant defense mechanism to scavenge excessive reactive oxygen species (ROS), exerting a short-term protective effect; however, under the background of persistent ischemia and reperfusion injury, long-term ROS accumulation leads to mitochondrial dysfunction, and the continuous high expression of SOD2 reflects a “compensatory but ineffective” antioxidant state, which further induces DNA damage, telomere instability, and activation of the p53/p21 pathway, ultimately promoting cell cycle arrest and the initiation of the senescence program (37). MXD1 antagonizes MYC to induce cell cycle arrest and trigger the senescence program; its upregulated expression in the AMI-related hypoxic and inflammatory microenvironment inhibits MYC-dependent transcriptional activity through competitive binding with MAX, thereby downregulating cell proliferation-related genes (such as the Cyclin and CDK families), directly causing cell cycle arrest in the G1 phase and providing a key prerequisite for the initiation of the senescence program, and its mediated sustained proliferation inhibition in cardiomyocytes and infiltrating immune cells further promotes the accumulation of senescent cells (5). GRN maintains the senescent phenotype through lysosomal function and inflammatory regulation; its significant upregulation in the AMI-induced inflammatory environment not only participates in immune regulation but also plays an important role in maintaining lysosomal function and cellular stress response, and abnormal GRN function can lead to impairment of the lysosome-autophagy pathway, thereby causing protein accumulation and disruption of intracellular homeostasis, while enhancing DNA damage response signals, and it also participates in the maintenance and amplification of the SASP network by regulating the secretion of inflammatory factors, promoting the persistence of the chronic inflammatory microenvironment at the tissue level (38). In summary, these four genes do not merely represent “non-specific stress responses” but correspond to different dimensions of the cellular senescence process, collectively supporting that the gene set identified in this study constitutes a multi-dimensionally integrated senescence regulatory network in the context of AMI, thereby enhancing its biological interpretability and not just reflecting a general inflammatory response.

In addition, this study also explored the potential role of AMI-related senescence processes in immunoregulation and inflammatory pathology. Subgroup analysis based on SRGs showed that anti-inflammatory cells were enriched in the low ASS, while the high ASS was mainly characterized by proinflammatory cell infiltration, with the most significant neutrophil infiltration. As typical proinflammatory cells, neutrophils can further exacerbate myocardial injury by releasing inflammatory mediators (39). Functional analysis revealed that the senescence-related group was significantly enriched in pathways such as the “TNF signaling pathway”, “neutrophil activation pathway”, and other inflammation-related pathways, supporting a close association between senescence-related signatures and immune/inflammatory activation. Moreover, our single-cell analysis, after rigorous Benjamini-Hochberg false discovery rate (FDR) correction for multiple comparisons, further confirmed that SRGs were mainly enriched in neutrophils. The results indicate that immune cells, particularly neutrophils, are closely associated with inflammatory activation and senescence-related transcriptional features in AMI. Importantly, the present findings are primarily correlative and do not establish whether the identified SRGs directly drive neutrophil recruitment or activation. It is also possible that neutrophil-derived inflammatory mediators and oxidative stress contribute, in turn, to senescence-associated transcriptional changes. This offers an additional perspective for understanding the relationship between immunosenescence-related signatures, inflammation, and AMI pathology (40).

This study also has certain limitations: First, although the study included a variety of datasets, the sample size was still insufficient due to the limitations of public database resources. In the future, it will be necessary to further expand the samples and supplement more single-cell and proteomics data to validate the model performance. Second, despite validation with mouse models and patient sequencing data, there is still insufficient clinical experimental evidence to fully confirm the diagnostic efficacy of the identified SRGs in real-world clinical samples. The cross-species and cross-compartment validation strategy used in this study should also be interpreted with caution. The human discovery and validation cohorts were derived from peripheral blood transcriptomic datasets, which primarily reflect leukocyte-associated inflammatory and senescence-related signals, rather than direct myocardial senescence. In contrast, the *in vivo* validation experiments involved infarct-zone cardiac tissues and peripheral blood leukocytes from mouse MI models. Although concordant dysregulation across these sample types supports the overall biological relevance of the identified genes in AMI, cardiac tissue and peripheral blood represent fundamentally distinct biological compartments, and myocardial expression patterns should not be assumed to directly mirror peripheral blood transcriptional changes. In addition, the senescence-related signatures identified in this study should not be interpreted as direct evidence of bona fide cellular senescence itself, because cells expressing senescence-associated genes are not necessarily truly senescent cells. Rather, these signatures more likely represent senescence-related or inflammation-associated transcriptional features in the context of AMI. Another important limitation is that the original 8-week mouse MI model mainly reflects a standard acute injury response in young adult animals and does not fully recapitulate the chronic and age-dependent senescence background typical of human AMI. To better address this issue, we further performed qPCR and western blot validation in 18-month-old mice with MI and age-matched sham controls, and observed concordant dysregulation of the identified genes in this aged cohort. Although these additional data strengthen the age-related relevance of our findings, they still do not fully capture the biological complexity of aging-associated human AMI. Moreover, we did not further validate the protein expression of these candidate genes in independent clinical peripheral blood samples, and the prognostic value of the ASS and stratification system has not yet been established through systematic longitudinal clinical follow-up.

Despite the above limitations, we believe that this study provides effective biomarkers for the diagnosis of AMI and lays a foundation for future clarification of the specific mechanism of senescence-related AMI, thereby providing new strategies for the development of novel diagnosis and treatment methods for AMI. Finally, we hope that the diagnostic model, classification system, and senescence-related score system constructed based on senescence will contribute to the early diagnosis, treatment, and personalized treatment of AMI.

## Conclusions

This study identified 13 AMI-related senescence genes and four core diagnostic ones by integrating multi-omics data. Novel models, including a diagnostic model, a patient stratification system, and a senescence-related score system, were constructed based on these SRGs, demonstrating favorable predictive performance for AMI. Mechanistically, immune cells, especially neutrophils, may be associated with inflammatory activation and senescence-related transcriptional programs in AMI. This study provides candidate biomarkers for AMI diagnosis, lays a foundation for clarifying the specific mechanism of senescence - related AMI, and is expected to contribute to the early diagnosis, treatment, and personalized management of AMI through the constructed models.

## Data Availability

The datasets presented in this study are deposited in public online repositories. The RNA sequencing data generated in this study have been deposited in the Gene Expression Omnibus (GEO) repository, accession number GSE318951. The publicly available transcriptomic datasets analyzed in this study were obtained from GEO under accession numbers GSE34198, GSE48060, GSE60993, and GSE29111. The proteomic dataset analyzed in this study is available in the iProX repository under accession number PXD062794. The senescence-related gene set used in this study was obtained from the CellAge database. The names of the repository/repositories and accession number(s) can be found in the article/**Supplementary Material**.
