# Supplementary material for: Identification and analysis of diagnostic senescence-related gene signatures for acute myocardial infarction based on multi-omics data and machine learning
Source: Front Immunol. 2026 Jun 5;17:1775092. doi: 10.3389/fimmu.2026.1775092 (PMC13278993; doi:10.3389/fimmu.2026.1775092)
Supplement: Supplementary file 1 [file DataSheet1.pdf]

Supplementary Information

Identification and analysis of diagnostic senescence-related gene signatures for acute myocardial infarction based on multi-omics data and machine learning

This file includes:
Figures S1 to S5
Tables S1 to S4

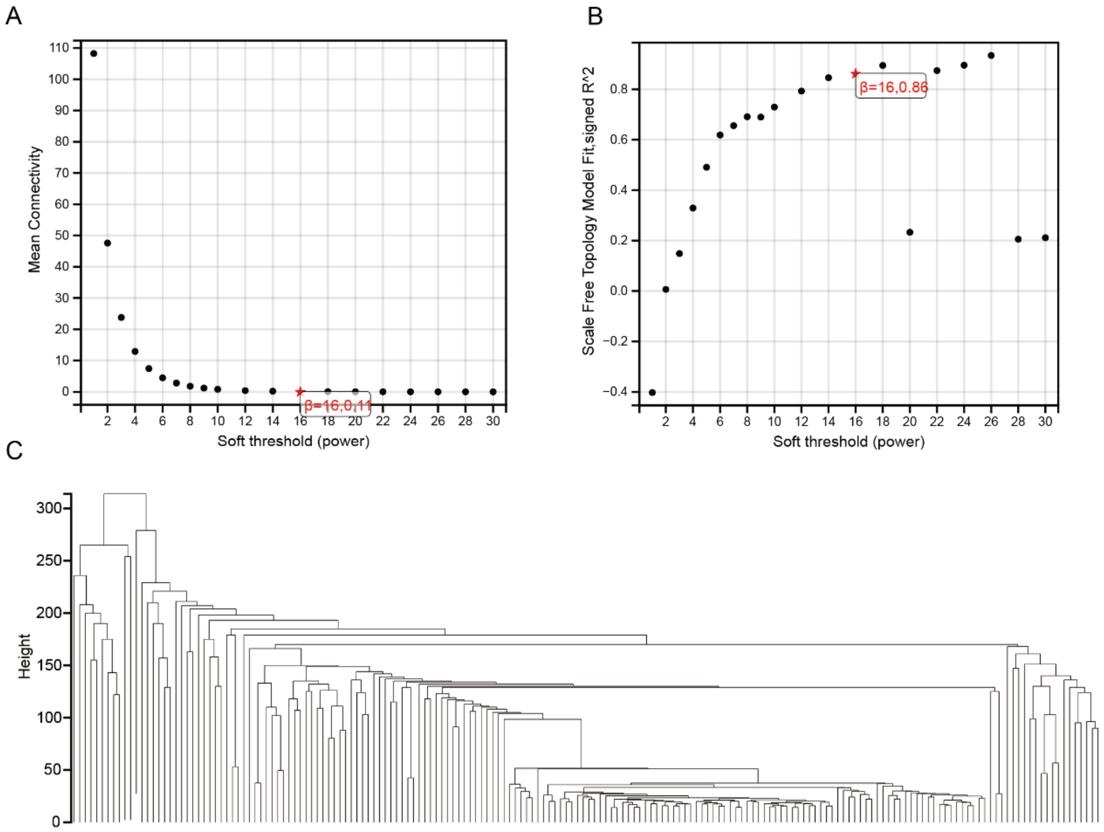

Figure S1. Election of the Soft Threshold (β) in WGCNA and Validation of Co-expression Network Construction.

(A) Curve showing the mean connectivity across different soft thresholds (power). At β = 16, the mean connectivity was 0.11. (B) Plot of the Scale Free Topology Model Fit-signed R^2 versus soft threshold. At β = 16, the model fit index reached R^2 = 0.86. (C) Hierarchical clustering tree of genes constructed based on the optimal soft threshold β = 16.

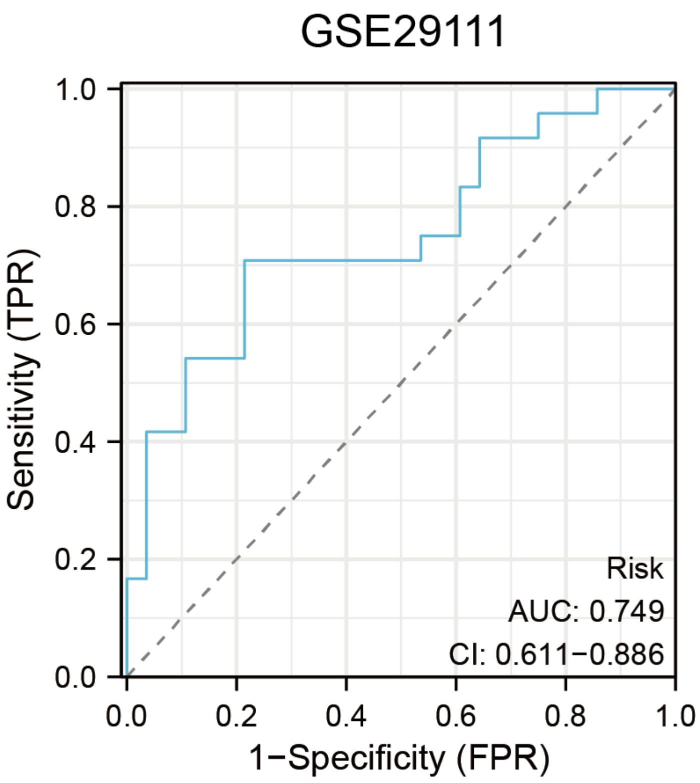

Figure S2. ROC curves of the diagnostic model validated in the external independent dataset GSE29111.

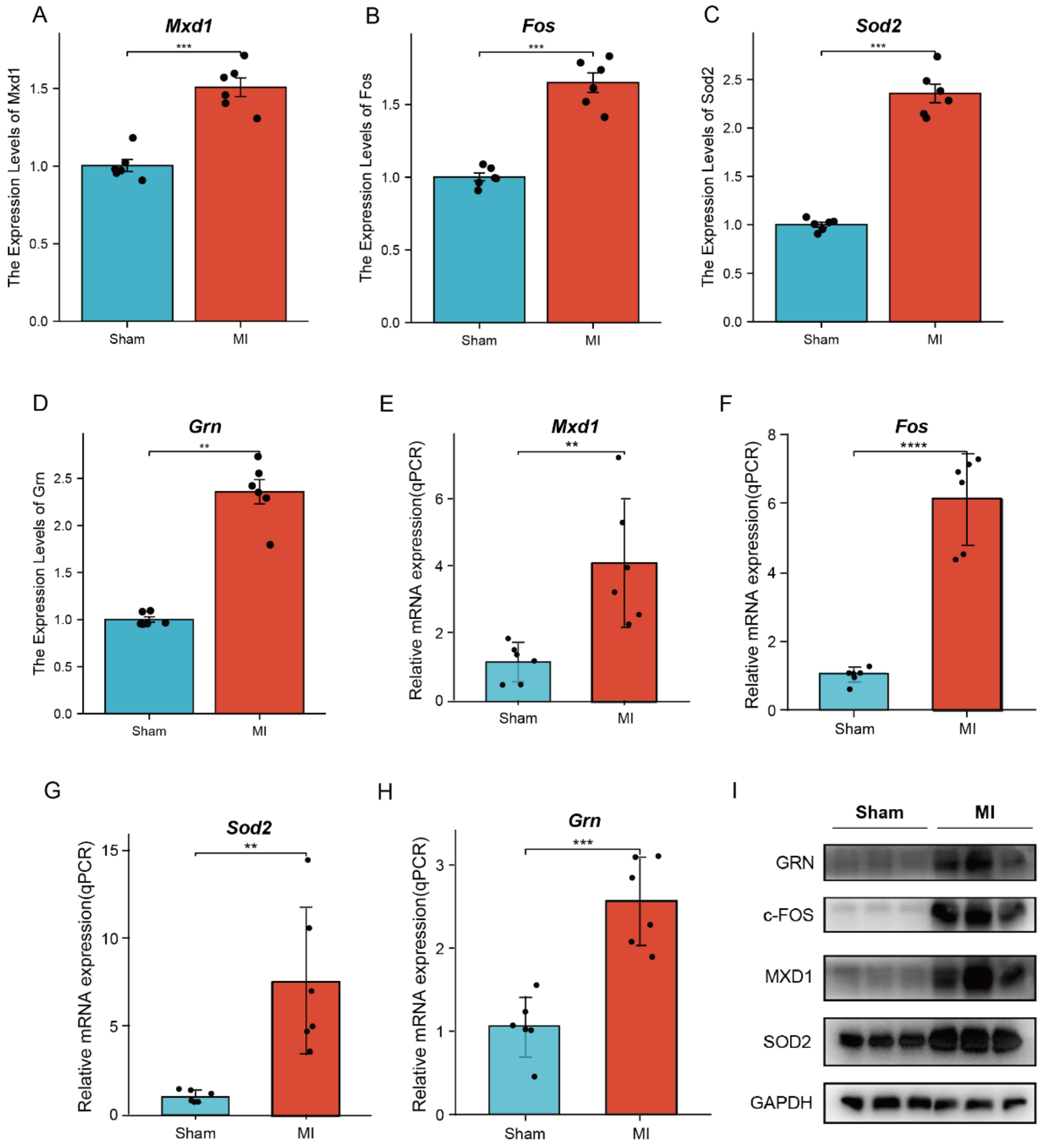

Figure S3. Supportive validation of the four diagnostic SRGs in mouse MI model. (A-D) Densitometric quantification of GRN, c-FOS, MXD1, and SOD2 protein levels normalized to GAPDH in infarct-zone cardiac tissues from 18-month-old sham and MI mice (n = 6 per group). Data are presented as mean ± SD. (E-H) Relative mRNA expression levels of *Mxd1*, *Fos*, *Sod2*, and *Grn* in peripheral blood leukocytes from 8-week-old sham and MI mice, as determined by qPCR. Data are presented as mean ± SD. **(I)** Representative western blots of GRN, c-FOS, MXD1, and SOD2 in infarct-zone cardiac tissues from 8-week-old sham and MI mice. Data are presented as mean ± SD.

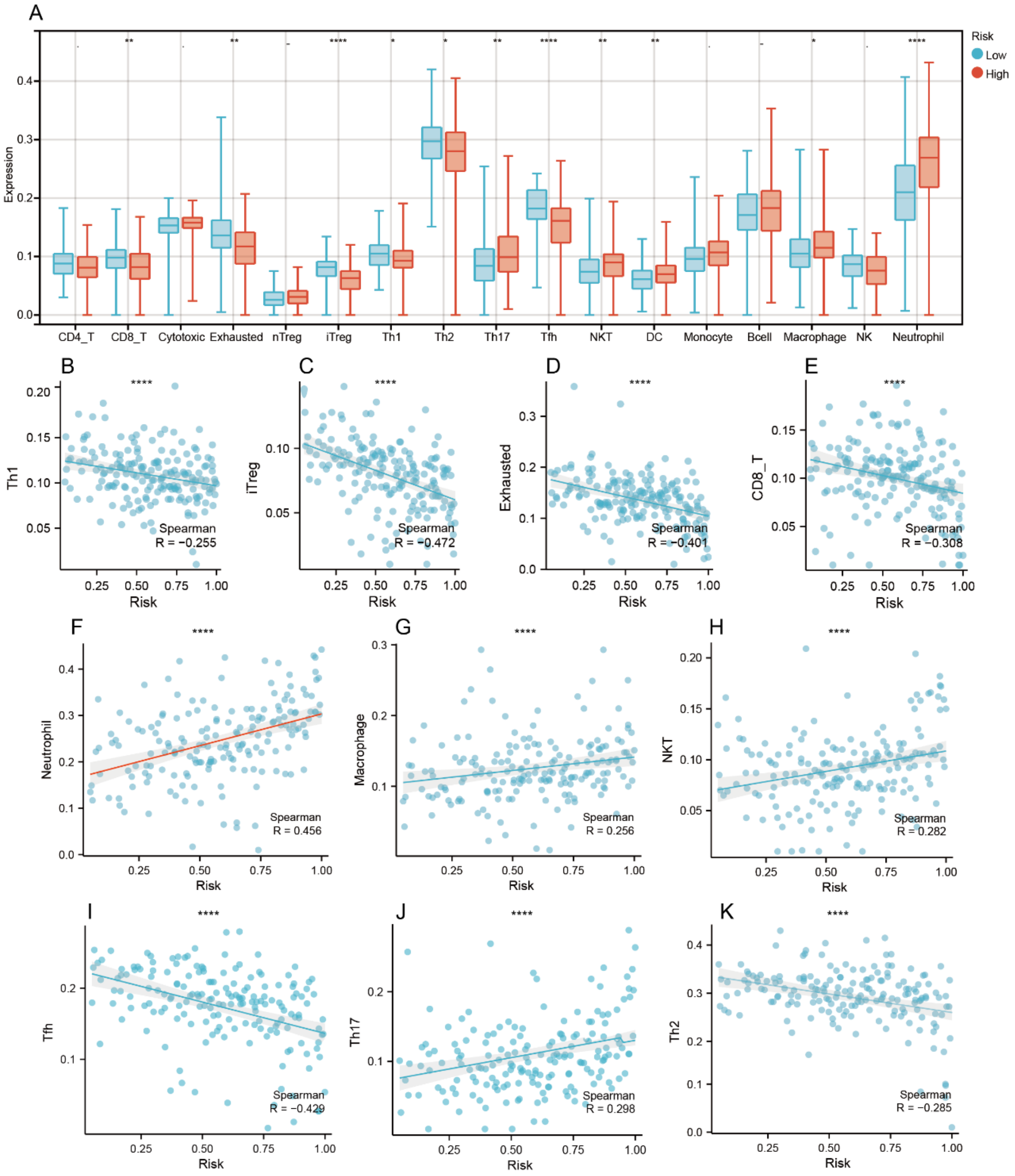

Figure S4. Immune cell infiltration analysis based on the SRG-based diagnostic model. (A) Boxplots comparing the infiltration levels of major immune-cell populations between high- and low-risk groups. Significance p-values adjusted for multiple comparisons. (B–K) Scatterplots showing the correlation between individual risk scores and the infiltration levels of the indicated immune-cell types (B–K).

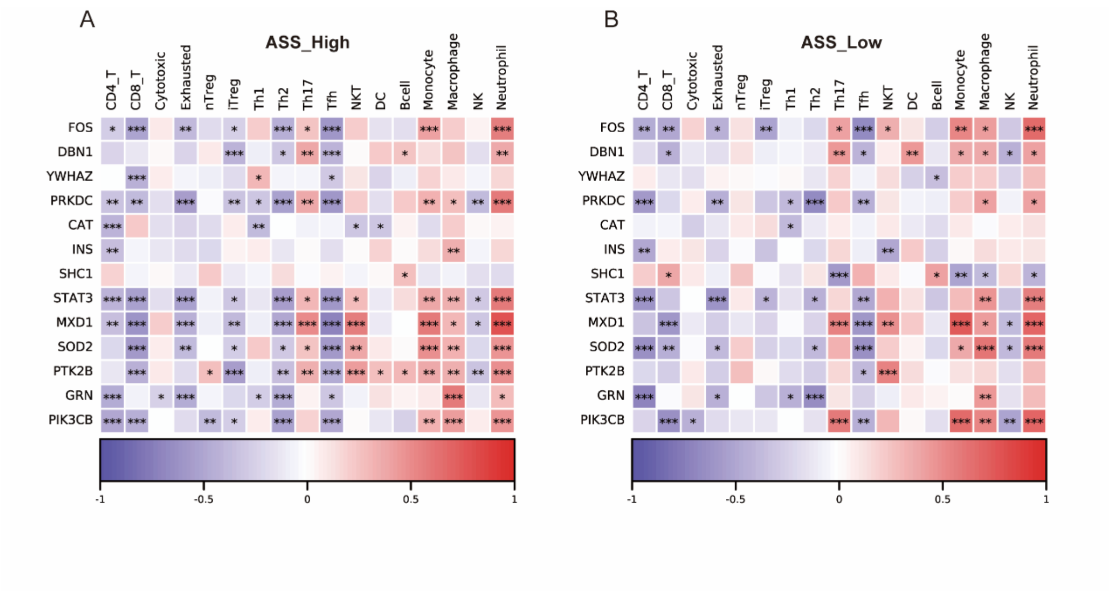

Figure S5. Correlation between SRGs expression and immune cell infiltration levels in high/low ASS groups. (A) Correlation heatmap between SRGs expression and immune-cell infiltration levels in the high-ASS subgroup. (B) Correlation heatmap between SRGs expression and immune-cell infiltration levels in the low-ASS subgroup.

Table S1. The senescence associated geneset from the CellAge website.

Senescence Associated Geneset						
GHR	JUND	CEBPB	LRP2	HMGB2	GHRHR	EEF1E1
GHRH	IL2	MXI1	AIFM1	MAP3K5	CTNNB1	EPS8
SHC1	PDGFB	TGFB1	UCHL1	TAF1	PSEN1	KCNA3
POU1F1	EGF	ERCC6	APP	LMNB1	DLL3	SIRT7
PROP1	IL2RG	STK11	APOE	SDHC	CDKN2A	SLC13A1
TP53	FOS	EP300	A2M	FOXO4	PPP1CA	SOCS2
TERC	PDGFRB	APTX	SNCG	HESX1	DBN1	TPP2
TERT	EPOR	PML	PRDX1	PIK3R1	NOG	TP53BP1
ATM	SST	GSK3B	PON1	BSCL2	ELN	SIRT3
PLAU	PRKCD	HTT	RELA	AGPAT2	ATR	NCOR2
ERCC2	PPARA	PRKCA	IL6	BMI1	UCP3	SUN1
ERCC8	RET	SSTR3	RGN	EEF1A1	ZMPSTE24	BAK1
WRN	PLCG2	HELLS	ATP5O	TFAP2A	TP63	IGFBP2
LMNA	PEX5	APOC3	RAD52	BDNF	UCP2	PYCR1
IGF1R	TCF3	EEF2	TOP3B	CREB1	POLB	TP73
TXN	PARP1	ERCC3	ERCC1	ATF2	GCLC	CNR1
KL	BRCA1	TERF1	SIRT1	TBP	GCLM	NFE2L2
E2F1	PIN1	PRKDC	HDAC1	APEX1	SIRT6	CDKN1A
PTPN11	PTEN	CAT	HSPA9	HBP1	BUB3	PDGFRA
NFKB2	CREBBP	ERCC5	GPX1	BUB1B	RAE1	PIK3CA
STAT5B	HIF1A	AR	GSR	PTGS2	PMCH	C1QA
STAT3	UBB	GTF2H2	GSS	HSPA8	MLH1	CDKN2B
STAT5A	RPA1	XRCC5	GSTA4	SIN3A	CSNK1E	EIF5A2
NRG1	BLM	PCNA	GSTP1	CDK1	STUB1	MIF
HDAC3	BCL2	FEN1	MT-CO1	TFDP1	PPM1D	DGAT1
GH1	S100B	FAS	HSPD1	DDIT3	CHEK2	MT1E
IL7R	VCP	TERF2	HSPA1A	POLA1	PCK1	FGF21
IGF1	POLG	XRCC6	HSPA1B	MAPT	ARHGAP1	HTRA2
IGF2	IGFBP3	POLD1	PCMT1	CTGF	CDC42	GSK3A
INS	HSP90AA1	BAX	MAPK8	HDAC2	ARNTL	NUDT1
NGF	NR3C1	RB1	YWHAZ	MAX	CLOCK	IKBKB
IRS1	EGR1	EMD	PTK2B	MXD1	HIC1	SQSTM1
PTPN1	VEGFA	GRB2	PTK2	MDM2	PAPPA	CDK7
IRS2	ABL1	FOXO3	IL7	SUMO1	ADCY5	GRN
AKT1	BRCA2	FOXO1	MAPK14	H2AFX	PPARGC1A	SERPINE1
PIK3CB	TOP2A	HSF1	FGFR1	HOXB7	GPX4	SPRTN
NGFR	TOP2B	XPA	SP1	HOXC4	UCP1	RICTOR
HRAS	NFKB1	MSRA	FLT1	JAK2	FGF23	CTF1
MYC	TOP1	RECQL4	JUN	ESR1	EFEMP1	TRAP1
EGFR	RAD51	SOD2	MED1	LEP	ERCC4	TRPV1
ERBB2	UBE2I	SOD1	MAPK9	LEPR	CETP	NFE2L1
INSR	TNF	FOXM1	MAPK3	NFKBIA	PPARG	IFNB1

NCOR1	PDPK1	COQ7	HMGB1	CLU	AGTR1	GDF11
NBN	CEBPA	CACNA1A	CCNA2	MTOR	CISD2	

Table S2. The primer sequences used for real-time PCR.

Gene	Forward Primer（5'—3'）	Reverse Primer（5'—3'）
Grn	TACACCACGGATCTCCTGACCAAG	GTTGAGGCGGCAGCAGGTATATC
Mxd1	CTGGAGCGGCGGGAGAGAG	TGCTACTGGTGCTGTTCTTCTTGG
Fos	ACCCTGAGCCCAAGCCATCC	CGGGAGGTCTCTGAGCCACTG
Sod2	TCCCAGACCTGCCTTACGACTATG	CTCCTCGGTGGCGTTGAGATTG
β-Actin	AAGGCCAACCGTGAAAAGAT	GTGGTACGACCAGAGGCATAC

Table S3. The antibodies used for Western Bolt.

Target antigen	Vendor or Source	Catalog #	Working concentration
MXD1 Polyclonal antibody	Proteintech	17888-1-AP	1:1000
Granulins rabbit pAb	Upingbio	YP-Ab-12520	1:1000
c-Fos Monoclonal antibody	Proteintech	66590-1-Ig	1:5000
SOD2 Monoclonal antibody	Proteintech	66474-1-Ig	1:5000
GAPDH Mouse mAb	Abclonal	AC002	1:5000

Table S4. Functional orientation of DEGs.

Gene	Primary Function	Role in Senescence
**FOS**	Immediate early transcription factor	Dual role
**DBN1**	Cytoskeletal organization	Dual role (weak evidence)
**MXD1**	MYC antagonist	Anti-senescence
**PTK2B**	Tyrosine kinase signaling	Dual role
**GRN**	Inflammation regulator	Dual role
**SOD2**	Mitochondrial antioxidant enzyme	Anti-senescence
**YWHAZ**	14-3-3 signaling protein	Dual role
**PRKDC**	DNA damage repair (NHEJ)	Anti-senescence
**CAT**	Antioxidant enzyme (catalase)	Anti-senescence
**INS**	Insulin signaling	Dual role
**SHC1**	Adapter protein (p66Shc isoform)	Pro-senescence
**STAT3**	Transcription factor (JAK/STAT pathway)	Pro-senescence
**PIK3CB**	PI3K-AKT signaling pathway	Pro-senescence
